# STAT2 Controls Colorectal Tumorigenesis and Resistance to Anti-Cancer Drugs

**DOI:** 10.3390/cancers15225423

**Published:** 2023-11-15

**Authors:** Mircea T. Chiriac, Zsuzsanna Hracsko, Christoph Becker, Markus F. Neurath

**Affiliations:** 1 Department of Medicine 1, Gastroenterology, Endocrinology and Pneumology, University Hospital Erlangen, University of Erlangen-Nuremberg, 91054 Erlangen, Germany; 2 Deutsches Zentrum Immuntherapie (DZI), University Hospital Erlangen, 91054 Erlangen, Germany; 3 Ludwig Demling Endoscopy Center of Excellence, University Hospital Erlangen, University of Erlangen-Nuremberg, 91054 Erlangen, Germany

**Keywords:** anti-cancer therapy, *Apc^Min/+^*, colorectal cancer mouse model, STAT2, tumoroid

## Abstract

**Simple Summary:**

Despite recent improvements in survival rates, colorectal cancer is still responsible for millions of premature deaths worldwide. A better understanding of the steps that lead from chronic inflammation to cancer progression could offer new therapeutic options and save many lives. The present study aimed to investigate the role of the transcription factor signal transducer and activator of transcription 2 (STAT2) in colorectal cancer by taking advantage of experimental mouse models and three-dimensional tumoroids. Our results indicate that STAT2 promotes colorectal cancer by various mechanisms and that anti-cancer drugs could easily kill tumor cells lacking STAT2. It is conceivable that future therapeutic strategies for colorectal cancer might try to mitigate the actions of STAT2 to improve the treatment outcome in affected patients.

**Abstract:**

Colorectal cancer (CRC) is a significant socioeconomic burden in modern society and is accountable for millions of premature deaths each year. The role of signal transducer and activator of transcription 2 (STAT2)-dependent signaling in this context is not yet fully understood, and no therapies targeting this pathway are currently being pursued. We investigated the role of STAT2 in CRC using experimental mouse models coupled with RNA-sequencing (RNA-Seq) data and functional assays with anti-cancer agents in three-dimensional tumoroids. *Stat2^−/−^* mice showed greater resistance to the development of CRC in both inflammation-driven and inflammation-independent experimental CRC models. In ex vivo studies, tumoroids derived from *Stat2^−/−^* mice with the multiple intestinal neoplasia (Min) mutant allele of the adenomatous polyposis coli *(Apc)* locus exhibited delayed growth, were overall smaller and more differentiated as compared with tumoroids from *Apc^Min/+^* wildtype (WT) mice. Notably, tumoroids from *Apc^Min/+^ Stat2^−/−^* mice were more susceptible to anti-cancer agents inducing cell death by different mechanisms. Our findings clearly indicated that STAT2 promotes CRC and suggested that interventions targeting STAT2-dependent signals might become an attractive therapeutic option for patients with CRC.

## 1. Introduction

Colorectal cancer (CRC) is the third most frequent cause of cancer-related deaths in both men and women, ranking second when numbers in both genders are combined [1,2]. There are roughly 150,000 new cases every year in the United States and two million worldwide, making CRC the third most prevalent cancer, not including skin tumors [1,2]. CRC frequently occurs as a lasting complication of inflammatory bowel disease (IBD) [3,4], a group of chronic, relapsing, and aggravating disorders in which the homeostasis of the gastrointestinal tract is being disrupted by aberrant immune responses against environmental and microbial assaults in genetically predisposed individuals [5,6]. Microbial products that interact with the intestinal epithelium trigger an immune response hallmarked by the secretion of copious amounts of pro-inflammatory cytokines, such as interferon (IFN), interleukin (IL)-1, IL-6, and tumor necrosis factor (TNF) [5,7]. Anti-inflammatory mediators, including IL-10 and transforming growth factor β are also produced to aid in coordinating the resolution phase of inflammation through various tissue repair and remodeling mechanisms, ultimately restoring homeostasis [8,9]. The disruption of this fragile balance between pro- vs. anti-inflammatory states, such as through the increased release of type I IFN, can result in excessive epithelial cell death. This, in turn, creates an environment that attracts more immune cells thus perpetuating the vicious cycle of chronic inflammation and ultimately leading to malignant transformation, cancer growth, and metastasis [3,4,10,11,12]. New insights provided by a better understanding of basic molecular mechanisms governing the interplay between tumor and non-tumor cells (i.e., infiltrating immune cells, cancer-associated fibroblasts, myofibroblasts, and blood vessels) have established the groundwork for innovative anti-cancer treatments, such as monoclonal antibodies/immune checkpoint inhibitors [13]. Nevertheless, not all patients respond, and in monotherapy regimens, the final goal, i.e., tumor-free survival is often not achieved. Assessment of anti-cancer drug resistance through the analysis of spatial structure modifications by genome-wide three-dimensional multi-omics approaches should help in gaining novel insights into the functional role of chromatin structure in the development of CRC and the resistance to therapy [14]. Furthermore, very promising platforms including the magnetic micro-/nano-robots have recently been demonstrated to possess great potential for targeted anti-cancer therapy [15].

More than 65 years ago, type I IFN was discovered by researchers studying the anti-viral response [16,17]. The mechanistic details of how type I IFN signaling occurs have been worked out during the early nineties [18,19] by identifying the first signal transducer and activator of transcription (STAT) members of the Janus kinase (JAK)-STAT family, namely, STAT1 and STAT2. As it turned out, JAK-STAT is one of a handful of pathways implicated in processes ranging from organismic development to cell proliferation, differentiation, and activation, from homeostasis to the regulation of cell death and inflammation, as well as the development and the spread of cancer [20]. Although it is arguably one of the most researched pathways, clinical applications relying on modulating type I IFN function have not yet become a part of the mainstream arsenal of currently used anti-cancer drugs. The underlying reasons may well be related to the extremely intricate processes induced by type I IFN/STAT signals. Upon engagement of the type I IFN receptor, the tyrosine kinases associated with the intra-cytoplasmatic tails of the receptor become trans-phosphorylated and further phosphorylate the intracellular tails of the receptors, thereby creating docking sites for STAT1 and STAT2. Once phosphorylated, STAT1 and STAT2 heterodimerize and become associated with their DNA-binding partner, interferon regulatory factor (IRF)9, to form the interferon-stimulated gene factor 3. This translocates to the nucleus and binds to hundreds of interferon-stimulated genes via interferon-stimulated response elements present in their promoters [21]. However, this canonical pathway by which STAT2-mediated type I IFN signals induce cell death and promote inflammation, as recently reported for experimental colitis [22], is only one possible outcome. It is by now well established that proliferation may also occur by engaging different partners (e.g., STAT1:STAT3) depending on cell type and the in vivo dynamics of the microenvironment.

It has been shown that the tumor growth in *Ifnar1^−/−^* mice was enhanced following the transplantation of syngeneic C3H melanoma K1735 cells, allogeneic 3LL carcinoma cells, or allogeneic B16F10 melanoma cells [23]. Similarly, *Ifnar1^ΔIEC^* mice that lacked IFN alpha receptor 1, specifically in the intestinal epithelial cells, exhibited increased tumor burden in the azoxymethane (AOM)–dextran sulfate sodium (DSS) model of CRC despite not developing spontaneous inflammation or increased severity in DSS colitis when compared with sufficient littermates [12]. In vitro studies using various cell lines indicated that STAT2 mediates the anti-tumor effects of type I IFN [24,25,26,27]. Whilst *Stat2^−/−^* mice do not develop spontaneous tumors for up to one year (our unpublished observations), transgenic mice lacking STAT2 but overexpressing type I IFN develop spontaneous medulloblastoma [28]. Furthermore, Gamero et al. reported for the first time that the absence of STAT2 led to decreased tumor formation in colitis-associated neoplasia in mice, which was accompanied by a decrease in STAT3 phosphorylation [29]. Moreover, the same group later showed that STAT2 blocks tumorigenesis in other experimental settings [30], drawing attention to the multifaceted nature of STAT2-mediated signals in tumorigenesis [10].

To better understand how STAT2 can impact CRC, in the present study, we aimed at investigating the contribution of STAT2-dependent signals to the development of CRC. We used a combination of experimental mouse models coupled with functional assays focusing on the effects of anti-cancer agents on tumoroids produced from wildtype (WT) and *Stat2^−/−^* mice with the multiple intestinal neoplasia (Min) mutant allele of the adenomatous polyposis coli *(Apc)* locus (*Apc^Min/+^* background). Our findings indicated that STAT2 exhibits a pathogenic contribution to the development of CRC and enhances the resistance to anti-cancer therapy.

## 2. Materials and Methods

### 2.1. Experimental Mouse Models of CRC

Experimental inflammation-driven CRC was induced in WT C57BL/6J (*n* = 11) and *Stat2^−/−^* mice (*n* = 14) on the C57BL/6J background (B6.129-*Stat2^tm1Shnd^*/J, JAX Strain #:023309, The Jackson Laboratory, Bar Harbor, ME, USA) [31] by a single intraperitoneal injection of AOM (Sigma-Aldrich, Schnelldorf, Germany). Mice subsequently received three cycles of DSS (MP Biomedicals, Eschwege, Germany) in the drinking water as previously described [32]. In this setting, AOM (10 mg/kg body weight) acts as a mutagen driving tumor development in the context of sustained inflammation induced by the administration of DSS (2%). Whereas inflammation dominates the picture in the first third of the model, tumors typically emerge during the second third and increase in number and size. During the final third of the experiment, inflammation becomes secondary after the cessation of DSS administration and tumor development reaches its maximum point. The high-resolution video mini-endoscopic system COLOVIEW (Karl Storz, Tuttlingen, Germany) was used to evaluate disease progression. Intestinal inflammation was assessed based on criteria previously defined for the modified murine endoscopic index of colitis severity (MEICS) which scores the following five parameters from 0 to 3: fibrin, granularity, stool, translucency, and vascularity [32]. Post-mortem, inflammation was additionally assessed by the shortening of the colon length and the measurement of expression levels of inflammation markers by quantitative polymerase chain reaction (*Ido1*, *S100a8*, *S100a9*, *Saa3*). *Stat2^−/−^* mice were backcrossed to C57BL/6J-*Apc^Min^*/J, JAX stock: #002020 (The Jackson Laboratory, Bar Harbor, ME, USA) [33,34] to generate *Apc^Min/+^ Stat2^−/−^* mice. *Apc^Min/+^* WT control mice (*n* = 10) and *Apc^Min/+^ Stat2^−/−^*mice (*n* = 13) were used for the sporadic model of CRC. In both models (AOM–DSS and *ApcMin*), tumor scoring was based on previously defined criteria [32]. Briefly, tumors observed during endoscopy were counted to obtain the overall number of tumors/mouse. Additionally, the size of each tumor was first graded as follows: grade 1 (very small but detectable tumor); grade 2 (tumor covering up less than one-eighth of the colonic circumference); grade 3 (tumor covering up less than a quarter of the colonic circumference); grade 4 (tumor covering up less than half of the colonic circumference); grade 5 (tumor covering up more than half of the colonic circumference). The tumor score was then calculated by adding the sizes of all tumors in a given mouse.

### 2.2. Generation and Assessment of Apc^Min/+^ Tumoroids

#### 2.2.1. Tumoroid Generation

Tumors were isolated from the colon of *Apc^Min/+^* WT (*n* = 5) and *Apc^Min/+^ Stat2^−/−^* (*n* = 6) mice as previously described [35]. Briefly, pre-incubation with 2 mM ethylenediaminetetraacetic acid in phosphate-buffered saline was carried out for one hour on ice before digestion with collagenase D (200 U/mL, Roche, Mannheim, Germany) and Dispase II (125 µg/mL, Thermo Fisher Scientific, Schwerte, Germany). The resulting cells were suspended in Cultrex Reduced Growth Factor Basement Membrane Extract, Type 2, Pathclear (Bio-Techne, Wiesbaden, Germany) and cultured in Advanced Dulbecco’s Modified Eagle’s Medium/F12 medium (Sigma-Aldrich, Schnelldorf, Germany or Thermo Fisher Scientific, Schwerte, Germany) supplemented with glutamine (2 mM, Thermo Fisher Scientific, Schwerte, Germany), HEPES, N-acetylcysteine (1 mM, Sigma-Aldrich, Schnelldorf, Germany), Penicillin-Streptomycin (100 U/mL and 100 µg/mL, Thermo Fisher Scientific, Schwerte, Germany), B-27 50×(Thermo Fisher Scientific, Schwerte, Germany), and epidermal growth factor (50 ng/mL, Thermo Fisher Scientific, Schwerte, Germany). Tumoroids were split once a week in a ratio of 1:4. The perimeter was determined as previously described [36].

#### 2.2.2. Assessment of the Effects of Anti-Cancer Drugs on Tumoroids

Tumoroids were either left untreated (vehicle control only) or were stimulated with the anti-cancer drugs 5-Fluorouracil (10 µM, 50 µM, 250 µM) or Cisplatin (10 µM, 25 µM, 100 µM), as indicated by the manufacturer (both Selleckchem, Darmstadt, Germany), i.e., dimethyl sulfoxide was used as a vehicle to dilute 5-Fluorouracil and water was used to dilute Cisplatin. We used bright field microscopy to follow the phenotypic characteristics of tumoroids (growth, budding, differentiation) in multiple wells focusing on at least 10 tumoroids/well.

To determine tumoroid viability, we used Methylthiazolyldiphenyl-tetrazolium bromide (MTT)-formazan (Sigma-Aldrich, Schnelldorf, Germany). In this assay, the mitochondrial respiration is evaluated by the reduction in the yellow-colored, water-soluble, MTT to dark purple-colored, water-insoluble, MTT-formazan in a reaction that is catalyzed by mitochondrial dehydrogenase and only occurs in viable cells [37,38]. Briefly, after cell death induction, MTT solution was added to the tumoroid culture to a final concentration of 500 µg/mL, and multi-well plates were further incubated for one hour at 37 °C. Then, the medium was discarded and 20 µL of 2% Sodium dodecyl sulfate solution (Carl Roth, Karlsruhe, Germany) in H_2_O was added to each well to solubilize the matrix for one hour at 37 °C. Subsequently, 80 µL of dimethyl sulfoxide (Sigma-Aldrich, Schnelldorf, Germany) was added and incubated for one hour at 37 °C to solubilize the reduced MTT-formazan precipitates. The absorbance was measured at 565 nm in an M200 plate reader (Tecan, Crailsheim, Germany). The effects of anti-cancer drugs are presented relative to the values obtained in unstimulated tumoroids which were set as 100% reference controls. Complimentary staining with propidium iodide and Hoechst (each at 5 µg/mL, both Thermo Fisher Scientific, Schwerte, Germany) was performed as previously described [37,38] and tumoroids were imaged using a fluorescence microscope (Leica, Wetzlar, Germany).

### 2.3. Immunofluorescence and TUNEL

Immunofluorescence staining of tumoroids was performed in suspension as recently described [39], with minor modifications. Briefly, tumoroids were washed twice with ice-cold phosphate-buffered saline, fixed for 30 min with 4% Paraformaldehyde, permeabilized with 0.2% Triton-X, and stained with primary antibodies against pSTAT3 followed by fluorescently labeled secondary antibodies. For mouse tissue, colon cross-sections were stained with primary antibodies against pSTAT3 followed by fluorescently labeled secondary antibodies. Nuclei were counterstained with 4′,6-diamidino-2-phenylindole (DAPI) or with Hoechst 33342 (Thermo Fisher Scientific, Schwerte, Germany). The DeadEnd Fluorometric TdT-mediated dUTP Nick-End Labeling (TUNEL) assay was used as indicated by the manufacturer (Promega, Walldorf, Germany). This assay measures the fragmented DNA of apoptotic cells by catalytically incorporating fluorescein-12-dUTP at 3′-OH DNA ends using a recombinant Terminal Deoxynucleotidyl Transferase which forms a polymeric tail. The fluorescein-12-dUTP-labeled DNA was visualized by confocal fluorescence microscopy (Leica, Wetzlar, Germany).

### 2.4. Western Blot

Proteins were isolated using either Radioimmunoprecipitation Assay buffer or Tissue Protein Extraction Reagent (both Thermo Fisher Scientific, Schwerte, Germany) supplemented with proteinase inhibitor and phosphatase inhibitor (Roche, Mannheim, Germany or Cell Signaling Technology, Leiden, The Netherlands) and Phenylmethylsulfonyl fluoride (Cell Signaling Technology, Leiden, The Netherlands). The lysates were sonicated on ice 3 times with 10 s pulses at 30% amplitude using a Branson Digital Sonifier 450 (Branson Ultrasonics Corp, Brookfield, CT). Proteins were denatured in Nu-Polyacrylamide Gel Electrophoresis Lithium Dodecyl Sulfate sample buffer (Thermo Fisher Scientific, Schwerte, Germany) or Laemmli sample buffer (Bio-Rad, Feldkirchen, Germany) with 50 mM Dithiothreitol (Bio-Rad, Feldkirchen, Germany) and were resolved on 4–15% Mini-PROTEAN TGX gels (Bio-Rad, Feldkirchen, Germany) and then transferred onto nitrocellulose membranes (Trans-Blot Turbo RTA Mini NC Kit) using the Trans-Blot Turbo Transfer System (all Bio-Rad, Feldkirchen, Germany). Non-specific binding was blocked by incubating membranes in 5% non-fat dry milk (Cell Signaling Technology, Leiden, The Netherlands) in 0.1% Tris-buffered saline-Tween-20 for one hour. Primary antibodies to pSTAT3, STAT3, cyclin D1, high mobility group box 1 (HMGB1), β-actin or GAPDH, and secondary horseradish peroxidase–conjugated antibodies were used as recommended by the manufacturer (Cell Signaling Technology, Leiden, The Netherlands). Supersignal West Pico Plus or Femto Max Substrate (Thermo Fisher Scientific, Schwerte, Germany) were used to detect chemiluminescence. For quantification purposes, the intensity of each band was first normalized to the intensity of the actin band. The resulting values obtained in tumor samples were then divided by the actin-normalized value of the non-tumor sample. The same strategy was applied for WT and STAT2-deficient mice. GAPDH was used for the normalization of pSTAT3 and STAT3 levels in non-tumor samples from *Apc^Min/+^* mice as indicated in the Supplementary Figure S2 legend.

### 2.5. Quantitative Real-Time Polymerase Chain Reaction

Total RNA was isolated using RNA extraction kits following the manufacturer’s instructions (Peqlab/VWR, Ismaning, Germany or Macherey-Nagel, Düren, Germany). Up to 1 µg was reverse-transcribed into cDNA using the SCRIPT reverse transcriptase (Jena Bioscience, Jena, Germany). Quantitative polymerase chain reaction was performed in duplicates on a real-time thermal cycler (Bio-Rad, Feldkirchen, Germany) using the SsoAdvanced Universal SYBR Green Supermix (Bio-Rad, Feldkirchen, Germany) and QuantiTect primers (Qiagen, Hilden, Germany) specific for mouse *Actb*, *Axin2*, *Ccnd1*, *Cd33*, *Ido1*, *Il6*, *Lgr5*, *Muc2*, *Prom1/Cd144*, *S100a8*, *S100a9*, and *Saa3*. Relative mRNA expression was calculated as (Potency (efficiency target gene;-[CT target gene])/(Potency (efficiency reference gene;-[CT reference gene])).

All statistical values presented in this study were analyzed with GraphPad Prism 9 (GraphPad Software, San Diego, CA, USA) using Welch *t*-test, Mann–Whitney or Spearman Correlation Coefficient as indicated in the Figure legends. Results are presented as mean with SEM. Significance levels for *p* values: *, <0.05; **, <0.01; ***, <0.001; ****, <0.0001.

## 3. Results

### 3.1. STAT2-Dependent Signaling Regulates Intestinal Inflammation and the Development of Inflammation-Driven Colorectal Tumors

**Figure 1:**
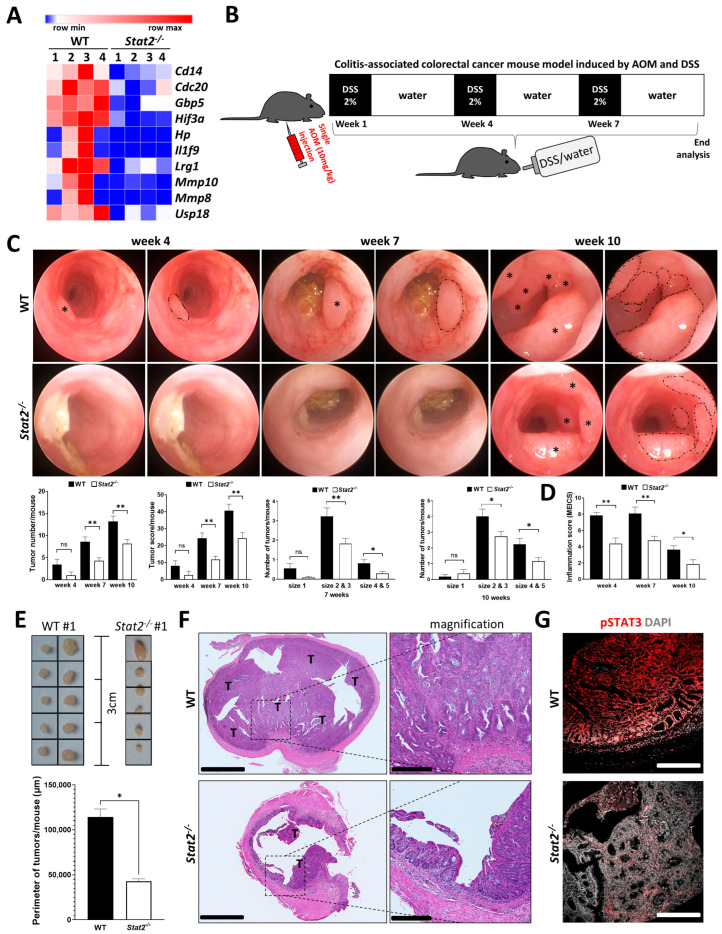
STAT2-dependent signaling controls inflammation during DSS-induced colitis and tumorigenesis in the AOM–DSS model of experimental CRC. (**A**) RNA-Seq data analysis was performed in colon tissue samples from WT and *Stat2^−/−^* mice (*n* = 4/group) from a DSS-induced colitis experiment. The original RNA-Seq data are deposited on ArrayExpress E-MTAB-12655. Heatmap focused on selected genes that have been previously associated with CRC (pathogenesis, metastasis, markers of poor prognostic); these transcripts were present among the topmost downregulated genes in the resistant *Stat2^−/−^* mice as compared with WT mice. The panel was created with the online matrix visualization and analysis software Morpheus (https://software.broadinstitute.org/morpheus, accessed on 6 October 2023). (**B**) Experimental model design of the AOM–DSS used in this study. (**C**) High-resolution mini-endoscopy pictures were taken at 4, 7, and 10 weeks after the initiation of the treatment. Individual tumors of various sizes are indicated by asterisks (left) and surrounded by dashed lines (right). Quantification of the results focusing on the tumor number, tumor score, and size distribution at 7 and 10 weeks. Scoring criteria are described in Materials and Methods. (**D**) The level of intestinal inflammation was scored based on five established criteria of the MEICS score as described in Materials and Methods. (**E**) The number and size of isolated tumors from one WT and one STAT2-deficient mouse can be appreciated macroscopically; quantification of perimeters of all tumors/mouse is displayed below. (**F**) Hematoxylin and eosin staining was used for the histologic assessment of colon sections; T indicates tumor. Magnifications focus on the areas within dashed lines. (**G**) Immunofluorescence staining of phosphorylated STAT3 in colon cross-sections. DAPI was used to stain nuclei. Abbreviations: AOM, azoxymethane; CRC, colorectal cancer; DSS, dextran sulfate sodium; ns, not significant; RNA-Seq, RNA-Sequencing; WT, wildtype. Scale bars: in (**F**), 1 mm for the overview and 250 µm for the inset; in (**G**), 250 µm. Statistics: Welch’s *t*-test for pairwise comparisons of WT vs. *Stat2^−/−^* in (**C**–**E**); mean with SEM is presented. * *p* < 0.05; ** *p* < 0.01.

Our group has recently identified a critical role of STAT2 in the development of intestinal inflammation in different mouse models of IBD. In that study, the RNA-sequencing (RNA-Seq) analysis of colon tissue samples from WT and *Stat2^−/−^* mice receiving DSS indicated that the deficiency in STAT2 resulted in the downregulation of many genes that are implicated in the pathogenesis of experimental DSS-induced colitis and IBD [22]. Furthermore, among the topmost downregulated transcripts in *Stat2^−/−^* mice, we found those of many genes that have been previously associated with CRC (pathogenesis, metastasis, markers of poor prognostic). Differences in the expression levels of ten such genes are presented side by side in WT and *Stat2^−/−^* mice (Figure 1A). This observation suggested that STAT2 signals might not only promote intestinal inflammation but could be involved in the pathogenesis of CRC through diverse mechanisms (Supplementary Figure S1). To further investigate this hypothesis, we took advantage of the AOM–DSS model. This model is particularly well suited to analyze inflammation-driven tumor initiation and progression as it can mimic IBD-associated CRC in patients [40,41]. For this purpose, we subjected WT and *Stat2^−/−^* mice to a single intraperitoneal administration of AOM followed by three cycles of DSS/water (Figure 1B). Our analysis indicated that tumors developed faster in WT mice compared with *Stat2^−/−^* mice, as indicated by their earlier appearance, increased numbers, and bigger overall sizes. The results were documented using high-resolution mini-endoscopy in living mice (Figure 1C, dashed lines on the right-side images represent the contour of individual tumors of varying sizes which were marked by asterisks on the left-side images). Quantification of the results focusing on tumor number, total tumor score, and the size distribution of small, medium, and large tumors at two different time points confirmed that WT mice developed a more severe disease as compared with *Stat2^−/−^* mice (Figure 1C). The DSS-induced inflammation was also more pronounced in the WT as compared with the *Stat2^−/−^* mice as indicated by the assessment of the colitis severity (MEICS score) at four, seven, and ten weeks after the initiation of the experiment (Figure 1D). After euthanizing the mice, we isolated the individual tumors from the surrounding non-tumor colon tissue. In some STAT2-deficient mice, a few bigger tumors could also be detected by the direct inspection of the intestine. However, most STAT2-deficient mice did not present big tumors and by adding up the perimeter of all tumors/mouse, significantly increased values were found in WT compared with *Stat2^−/−^* mice (Figure 1E). Histologic analysis of colon sections confirmed the increased number of tumors in WT mice as compared with *Stat2^−/−^* animals (Figure 1F, T indicates tumor, higher magnification is presented in the inset). Staining of colon sections from WT and *Stat2^−/−^* mice receiving AOM–DSS indicated that the levels of activated STAT3, a well-known promoter of CRC, were downregulated in samples from the less susceptible *Stat2^−/−^* strain (Figure 1G). Taken together, these results indicated that STAT2 regulates intestinal inflammation and inflammation-driven tumorigenesis.

### 3.2. STAT2 Drives Tumorigenesis Independent of Intestinal Inflammation in Apc^Min/+^ Mice

**Figure 2:**
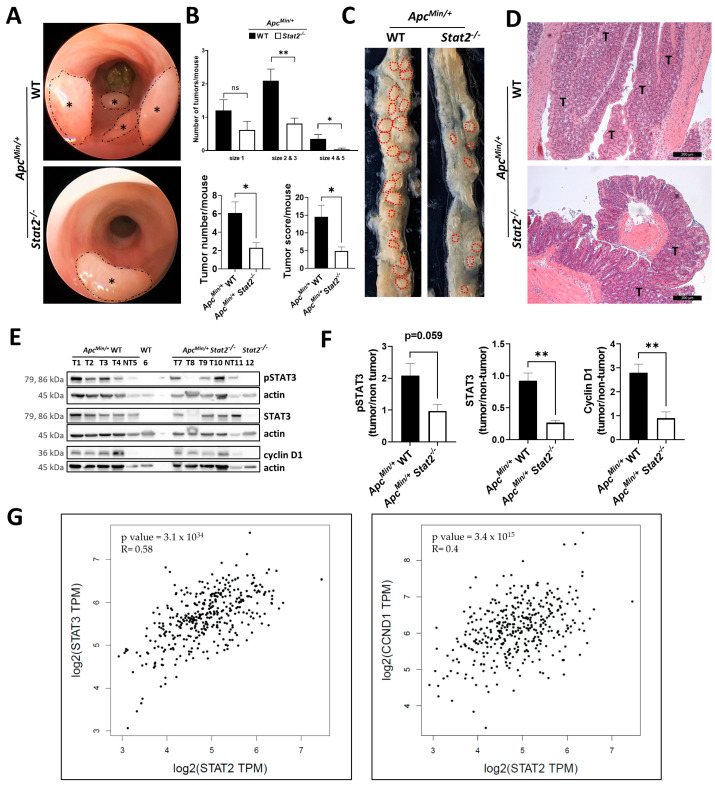
Tumor development in the *Apc^Min/+^* model is dependent on STAT2. (**A**) *Apc^Min/+^Stat2^−/−^* mice were generated by crossing *Stat2^−/−^* to *Apc**^Min/+^*** mice. High-resolution mini-endoscopy images of tumor development in the *Apc^Min/+^* model. Asterisks indicate and dashed lines surround individual tumors. (**B**) Quantification of total tumor numbers, distribution according to size, and the overall tumor score. Scoring criteria are described in Materials and Methods. (**C**) Tumors visible by the macroscopic inspection of the intestine are indicated by dashed lines. (**D**) Hematoxylin and eosin staining served as the histologic visualization of tumors under the bright field microscope; T indicates tumor. (**E**) Western blot of protein extracts obtained from the tumor (T1-T4 and T7-T10) and non-tumor (NT5 and NT11) tissue of *Apc^Min/+^* WT and *Apc^Min/+^Stat2^−/−^* mice, respectively. Protein extracts from WT (lane 6) and *Stat2^−/−^* (lane 12) mice that were not backcrossed to *Apc^Min/+^* mice served as additional controls. Levels of phosphorylated STAT3, STAT3, and cyclin D1 were detected and levels of actin were used as protein loading control. (**F**) Quantification of the normalized levels (described in Materials and Methods) of signal intensities from panel E. (**G**) Correlations between expression levels of *STAT2* and levels of *STAT3* and *CCND1* in CRC samples from a publicly available database (http://gepia.cancer-pku.cn/, accessed on 6 October 2023). Abbreviations: *Apc^Min/+^* mice with the multiple intestinal neoplasia (Min) mutant allele of the adenomatous polyposis coli (*Apc*) locus; CCND1, cyclin D1; ns, not significant. Scale bars in (**D**), 200 µm. Statistics: Welch’s *t*-test, for pairwise comparisons of *Apc^Min/+^* WT vs. *Apc^Min/+^Stat2^−/−^* in (**B**,**F**); mean with SEM is presented. Spearman Correlation Coefficient in (**G**). The uncropped bolts are shown in Supplementary Materials Figure S6. * *p* < 0.05; ** *p* < 0.01.

We next wondered whether the role of STAT2 in promoting colorectal tumorigenesis goes beyond its ability to regulate inflammation. To find out the answer to this question, we conducted additional experiments in the *Apc^Min/+^* mouse model of spontaneous colorectal carcinogenesis. This model in which multiple intestinal tumors develop, simulates human familial adenomatous polyposis and colorectal tumors [33,34]. Accordingly, *Stat2^−/−^* mice were backcrossed to *Apc^Min^* heterozygous mice, and the development of tumors was followed up by mini-endoscopy. The mice exhibited no signs of inflammation in the endoscopic evaluation (scores lower than 3 out of 15 points are considered to represent no inflammation, Supplementary Figure S2A). The lack of inflammation was confirmed by the fact that there was no colon length shortening at the end of the experiment (Supplementary Figure S2B). Similarly, no difference was observed in levels of inflammation markers as detected via quantitative polymerase chain reaction in samples collected at the end of the experiment (Supplementary Figure S2C). These results ensured that the *Apc^Min/+^* model is appropriate for investigating the inflammation-independent oncogenic role of STAT2 in these mice. As expected, *Apc^Min/+^* WT mice started to develop tumors at around the age of 10 weeks. In contrast, *Apc^Min/+^ Stat2^−/−^* mice developed fewer tumors at later time points (Figure 2A; asterisks indicate individual tumors that are surrounded by dashed lines). Quantification of the results confirmed the endoscopic evaluation by unveiling the fact that *Apc^-Min/+^* WT mice had more numerous tumors of bigger size and a higher total tumor score compared with *Apc^Min/+^ Stat2^−/−^* mice (Figure 2B). The difference was readily evident after euthanizing the mice and inspecting the dissected colons (Figure 2C; dashed lines mark the contour of individual tumors). Histologic analysis of colon sections revealed the higher penetrance of tumors in the *Apc^Min/+^* WT mice compared with the *Apc^Min/+^ Stat2^−/−^* mice (Figure 2D, T indicates tumor). Despite the fact that levels of inflammation markers did not differ between the strains (see above), the levels of the typical nuclear factor kappa B (NF-κB)-target gene *Il6* were increased in *Apc^Min/+^* WT as compared with Apc^Min/+^ Stat2^−/−^ mice, suggesting that a functional STAT2 might act in concert with NF-κB in this context (Supplementary Figure S2D). Tumor (samples T1-T4 and T7-T10) and non-tumor (samples T5 and T11) tissue was isolated from these mice and protein extracts were subjected to Western blot analysis. We investigated the levels of pSTAT3, STAT3, and cyclin D1 and used actin as a loading control for protein level normalization. Samples from WT (sample 6) and *Stat2^−/−^* (sample 12) mice without the *Apc^Min/+^* mutation served as additional controls (Figure 2E). The results indicated that pSTAT3 was increased in the tumor vs. non-tumor tissue from *Apc^Min/+^* WT compared with the *Apc^Min/+^ Stat2^−/−^* mice. Similarly, levels of STAT3 and its downstream target, cyclin D1, a promoter of CRC were increased. Notably, in non-tumor tissue samples, the levels of pSTAT3 and STAT3 were lower in WT as compared with *Stat2*-deficient *Apc^Min/+^* mice (Supplementary Figure S2E). Taken together, the results of this set of experiments indicated that STAT2-dependent signals can also promote inflammation-independent tumorigenesis in mice.

The relevance of our findings for CRC was further supported by the analysis of publicly available data from the Gene Expression Profiling Interactive Analysis [42] (http://gepia.cancer-pku.cn/, accessed on 6 October 2023). This online-based platform allows users to perform correlation analysis of genes in various cancer forms according to the classification of The Cancer Genome Atlas program. We found significant correlations between expression levels of *STAT2* and those of *STAT3* and cyclin D1, respectively, in tumor samples from colon and rectum adenocarcinoma (Figure 2G).

### 3.3. Tumoroids Derived from Apc^Min/+^ Stat2^−/−^ Mice Proliferate Slower, Remain Smaller, and Become More Differentiated Compared with Tumoroids Derived from Apc^Min/+^ WT Mice

**Figure 3:**
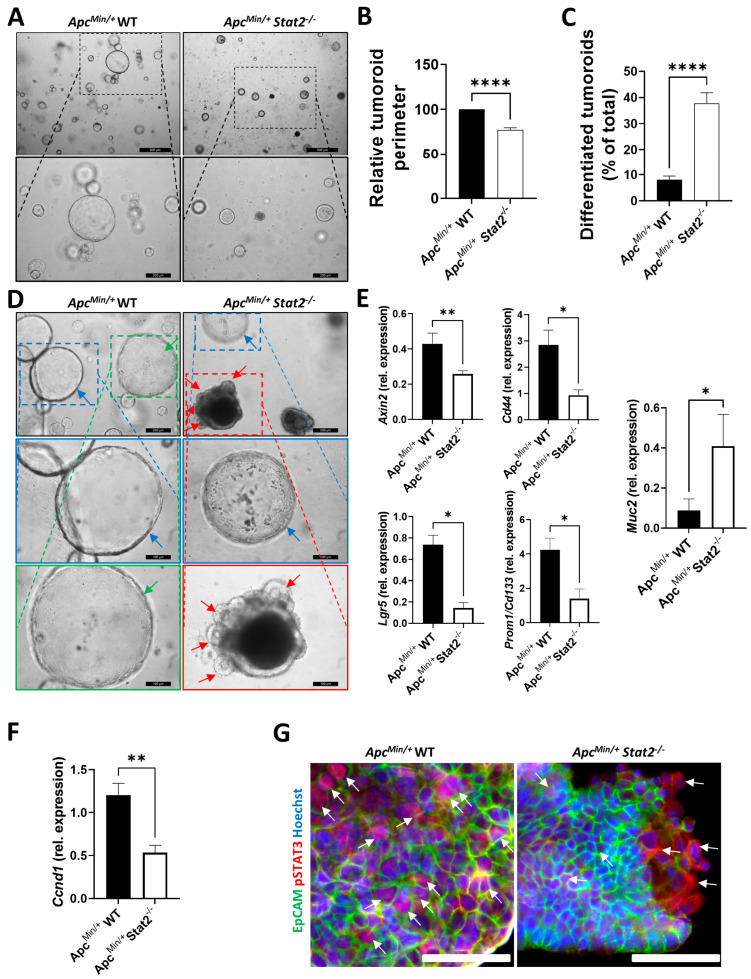
*Apc^Min/+^* tumoroids lacking STAT2 grow slower and become more differentiated. (**A**) Representative light microscopy images were taken two days after three-dimensional tumoroids produced from *Apc^Min/+^* WT and *Apc^Min/+^ Stat2^−/−^* mice had been passaged. Magnifications below focus on individual tumoroids as indicated by the dashed lines in the pictures above. (**B**) Perimeters of 50 tumoroids/group were measured and values from *Apc^Min/+^ Stat2^−/−^* were then divided by the values of the corresponding *Apc^Min/+^* WT tumoroids to obtain the relative tumoroid perimeter. (**C**,**D**) Differentiation was followed in more than 25 tumoroids/group. Thickening of the tumoroid wall, darkening of the tumoroid, accumulation of dead cells in the middle, or cell shedding as well as tumoroid budding served as criteria for assessing the differentiation status in tumoroids. In (**D**), blue or green arrows indicate less differentiated tumoroids and red arrows indicate highly differentiated tumoroids. Magnifications presented in the two lowest rows focus on individual tumoroids as indicated by the dashed lines in the pictures above. (**E**) Expression levels of epithelial stem cell/cancer stem cell markers (*Axin2*, *Cd44*, *Lgr5*, *Prom1/Cd133*) and the differentiation marker *Muc2* assessed by quantitative polymerase chain reaction relative to *Actb*. (**F**) Levels of *Ccnd1*, which controls the proliferation of intestinal epithelial cells, were measured by quantitative polymerase chain reaction relative to *Actb*. (**G**) Staining of pSTAT3 in tumoroids is indicated by arrows. EpCAM served as a specific marker for intestinal epithelial cells and Hoechst was used to stain nuclei. Abbreviations: Ccnd1, cyclin D1. Scale bars: in (**A**), 500 µm in the upper panels and 200 µm in the lower panels; in (**D**), 200 µm in the upper panels and 100 µm in the middle and lower panels; in (**G**), 50 µm. Statistics: Welch’s *t*-test in (**B**,**C**) and Mann–Whitney test in (**E**,**F**); mean with SEM is presented. * *p* < 0.05; ** *p* < 0.01; **** *p* < 0.0001.

Taken together, the analysis of RNA-Seq data coupled with results from our in vivo experimental CRC models indicated that STAT2 confers increased susceptibility to CRC in various settings relying on distinct mechanisms, i.e., inflammation-dependent (AOM–DSS) and inflammation-independent (*Apc^Min/+^*). To further understand whether STAT2-mediated signals within the epithelial cell compartment control tumorigenesis, we isolated individual tumors from *Apc^Min/+^* WT and *Apc^Min/+^ Stat2^−/−^* mice and cultured them as three-dimensional tumoroids. This model allows for the specific investigation of epithelial cell functions since non-epithelial cells do not survive in this setting. Although tumoroids of both strains could be easily produced, a difference in the growth dynamics became rapidly apparent. Specifically, *Apc^Min/+^* WT tumoroids grew faster and bigger as indicated by representative microscopy pictures taken a couple of days after freshly passaging tumoroids (Figure 3A; the lower panels focus on individual tumoroids from images presented above) and the assessment of the tumoroid perimeter (Figure 3B). Whereas all tumoroids grew further, *Apc^Min/+^ Stat2*^−/−^ remained smaller and we could clearly observe a higher frequency of tumoroids presenting signs of differentiation (e.g., thickening of the wall, darkening of the tumoroid, accumulation of dead cells in the middle, cell shedding, budding) in *Apc^Min/+^ Stat2^−/−^* as compared with *Apc^Min/+^* WT tumoroids (Figure 3C). Using representative microscopy images, we designated undifferentiated tumoroids by blue or green arrows and indicated differentiated, budding tumoroids by red arrows (Figure 3D; the lowest two rows of panels focus on individual tumoroids from images presented in the first row). This difference observed by microscopy could be further substantiated by the results of quantitative polymerase chain reaction. We found higher levels of epithelial stem cell/cancer stem cell markers (e.g., *Lgr5*, *Axin2*, *Cd44*, *Prom1/CD133*) in the tumoroids derived from *Apc^Min/+^* WT as compared with *Apc^Min/+^ Stat2^−/−^* mice (Figure 3E). In contrast, the levels of *Muc2*, the differentiation marker of goblet cells, were lower in the more undifferentiated *Apc^Min/+^* WT as compared with *Apc^Min/+^ Stat2^−/−^* tumoroids (Figure 3E). Furthermore, levels of cyclin D1, the downstream target of STAT3 which was upregulated in the tumor vs. non-tumor tissue from *Apc^Min/+^* WT as compared with *Apc^Min/+^ Stat2^−/−^* mice (refer to Figure 2E,F), were upregulated in *Apc^Min/+^* WT as compared with *Apc^Min/+^ Stat2^−/−^* tumoroids, strengthening the idea of an epithelial cell-specific contribution of STAT2 in the proliferation of *Apc^Min/+^* tumor cells (Figure 3F). Supporting a possible implication of STAT2-dependent epithelial-derived activity of STAT3 in this setting, higher levels of pSTAT3 were present in tumoroids derived from *Apc^Min/+^* WT as compared with *Apc^Min/+^ Stat2^−/−^* mice (Figure 3G, arrows).

### 3.4. Tumoroids Derived from Apc^Min/+^ Stat2^−/−^ Mice Are Highly Susceptible to Killing by Anti-Cancer Agents

**Figure 4:**
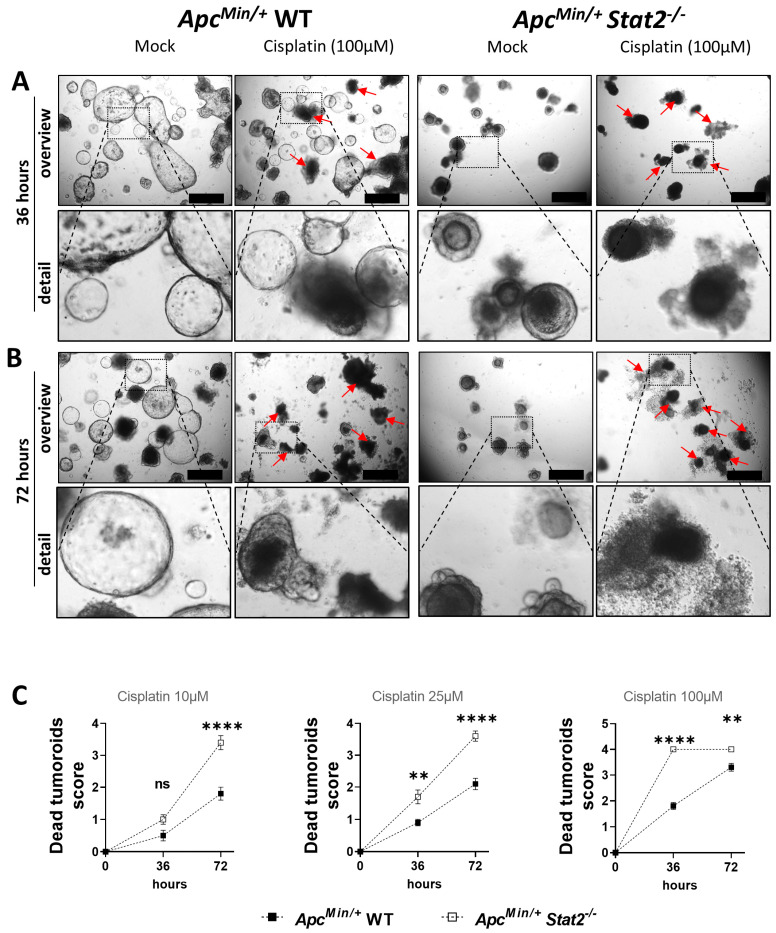
Cisplatin is highly effective in killing *Apc^Min/+^* tumoroids in a dose- and time-dependent manner, especially in cells lacking STAT2. (**A**,**B**) Light microscopy was used to follow the effect of 100 µM Cisplatin in *Apc^Min/+^* WT and *Apc^Min/+^ Stat2^−/−^* mice at (**A**) 36 h and (**B**) 72 h. Red arrows in A and B indicate highly differentiated/dead tumoroids. (**C**) The level of cell death in tumoroids that have been stimulated with three different doses of Cisplatin was assessed by counting the number of dead tumoroids in multiple wells/condition under the light microscope. Only living tumoroids as of day zero were considered and their faith over the next 72 h is reflected by the graphs at 36 and 72 h in (**C**). Abbreviations: ns, not significant. The mean with SEM is presented. ** *p* < 0.01; **** *p* < 0.0001.

The realization that many *Apc^Min/+^ Stat2^−/−^* become increasingly differentiated over time and some of them die, made us wonder whether cell death in these tumoroids could be accelerated by anti-cancer drugs since these agents more potently induce cell death in differentiated as compared with highly aggressive, undifferentiated, cancerous cells. To test this hypothesis in our setting, tumoroids derived from *Apc^Min/+^* WT and *Apc^Min/+^ Stat2^−/−^* mice were grown for up to one week after being passaged. One day after the medium was changed, Cisplatin, a first-line anti-cancer drug used in many cancers including CRC, was added. As indicated by microscopy pictures obtained 36 h after the beginning of Cisplatin treatment, some of the tumoroids derived from *Apc^Min/+^* WT mice showed signs of differentiation or died (red arrows). However, under the same conditions, there was a higher number of tumoroids derived from *Apc^Min/+^ Stat2^−/−^* that underwent differentiation or died (Figure 4A, insets represent detailed aspects of the areas indicated by the dashed line in the picture above). The effect of Cisplatin was even more pronounced after 72 h, and again, the numbers were higher (red arrows) in tumoroids derived from *Apc^Min/+^ Stat2^−/−^* as compared with *Apc^Min/+^* WT mice (Figure 4B, insets represent detailed aspects of the areas indicated by the dashed line in the picture above). Stimulations with three different doses of Cisplatin revealed the fact that higher doses and longer incubation times resulted in better killing which was significantly more pronounced in tumoroids derived from *Apc^Min/+^ Stat2^−/−^* as compared with *Apc^Min/+^* WT mice (Figure 4C).

**Figure 5:**
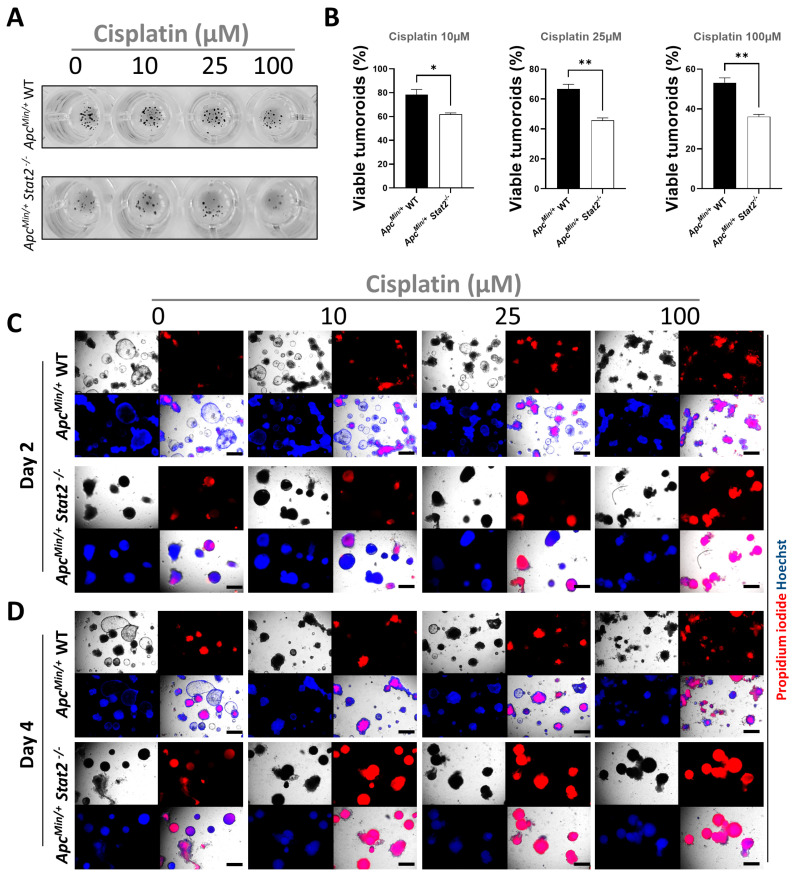
*Apc^Min/+^ Stat2^−/−^* are exquisitely sensitive to the effects of Cisplatin as revealed by the early cessation of mitochondrial activity and almost absolute cell death after 48 h of stimulation. (**A**,**B**) The MTT-formazan assay was used to investigate the effects of Cisplatin stimulation on the mitochondrial activity of tumoroids derived from *Apc^Min/+^ Stat2^−/−^* and *Apc^Min/+^* WT mice. (**A**) The presence of formazan precipitates which is proportional to the mitochondrial activity of living cells can be readily observed macroscopically by appreciating the black spots in the treated wells. The concentrations of the anti-cancer drug used in the assay are indicated above the images. (**B**) Quantification of the results from the MTT-formazan assay after 48 h of stimulation with Cisplatin is presented relative to unstimulated control wells for which viability was set as a reference at 100%. (**C**,**D**) Induction of cell death by three different doses of Cisplatin was evaluated by fluorescence microscopy after co-staining of *Apc^Min/+^ Stat2^−/−^* and *Apc^Min/+^* WT tumoroids with propidium iodide (red staining—dead cells) and Hoechst (blue staining—dead as well as living cells) at an early (two days) and a late (four days) time point. For each condition, a total of three pictures were taken (bright field, propidium iodide, and Hoechst), and merging these three individual channels produced the lower right image of each panel. The increased presence of magenta in these merged channels indicates higher rates of killed tumoroids (e.g., the lowest row at day 4, all *Apc^Min/+^ Stat2^−/−^* are dead, irrespective of the Cisplatin dose). Scale bars, 500 µm in (**C**,**D**). Statistics: Welch’s *t*-test in (**B**); mean with SEM is presented. * *p* < 0.05; ** *p* < 0.01.

To expand these observations, we employed complementary methods focusing on the assessment of cellular viability (MTT) and cell death (propidium iodide/Hoechst staining) in the multi-well plate setting as previously described [37,38]. Tumoroids were cultured and stimulated as described above. Forty-eight hours post-stimulation, MTT was added and the level of dark formazan precipitates, which is a measure of mitochondrial activity in living cells was first inspected macroscopically. The higher amount of dark spots is proportional to the number of living cells (Figure 5A). The measurement of the absorbance levels of solubilized formazan precipitates from those experiments indicated that increasing the dose of Cisplatin abolished mitochondrial function more potently and that *Apc^Min/+^ Stat2^−/−^* tumoroids were affected significantly more as compared with *Apc^Min/+^* WT tumoroids (Figure 5B). In complementary experiments, propidium iodide and Hoechst were simultaneously added to the tumoroids, and cell death was evaluated by fluorescence microscopy. In this assay, propidium iodide indicates dead cells while Hoechst stains all cells by its ability to bind DNA in both, living and dead cells. The results confirmed the dose- and time-dependent induction of cell death by Cisplatin and the fact that tumoroids derived from *Apc^Min/+^ Stat2^−/−^* mice were significantly more susceptible (virtually all STAT2-deficient tumoroids were dead by day 4) to its actions as compared with those derived from *Apc^Min/+^* WT mice (Figure 5C,D).

Cisplatin, the first FDA-approved platinum compound for cancer treatment, induces cell death mainly by cross-linking DNA after interacting with purines thus blocking cell replication and DNA repair mechanisms [43]. To examine how STAT2 might interfere with the Cisplatin-induced DNA damage response in our model, we first assessed the levels of HMGB1 in tumoroid culture supernatants. Being involved in the regulation of various DNA repair mechanisms and acting as an alarmin when secreted by dying cells, HMGB1 is being used as an indirect marker of DNA damage. HMGB1 was found in tumoroids of Cisplatin-stimulated *Apc^Min/+^ Stat2^−/−^* but was not observed in Cisplatin-stimulated *Apc^Min/+^* WT tumoroids or any unstimulated tumoroids (Supplementary Figure S3A). TUNEL staining results provided further evidence for the fact that STAT2 signals mediate resistance to Cisplatin treatment by reducing the DNA fragmentation process, which can be an indicator of DNA damage (Supplementary Figure S3B, arrows indicate TUNEL^+^ cells).

To investigate whether STAT2 interferes with cell death induced by anti-cancer drugs triggering other mechanisms, we performed additional experiments focusing on 5-Fluorouracil. The anti-cancer properties of this drug, which is also often used as a prime-line medication in patients with CRC, mainly rely on its ability to interfere with the synthesis of pyrimidine thymidylate, a building block precursor of DNA without which rapidly dividing cancer cells undergo thymineless death [44]. Comparable to the observations made with Cisplatin, 5-Fluorouracil induced cell death in a dose- and time-dependent manner as indicated by microscopy pictures taken 36 and 72 h after the beginning of stimulation. Once again, the effects were significantly more pronounced in tumoroids derived from *Apc^Min/+^ Stat2^−/−^* as compared with *Apc^Min/+^* WT mice (Supplementary Figure S4A,B, insets in the panels below represent detailed aspects of the areas indicated by the dashed lines in the pictures above; red arrows indicate tumoroids with differentiation signs/dead tumoroids). Stimulations with three different doses of 5-Fluorouracil revealed the fact that higher doses and longer incubation times resulted in more killing which was most pronounced in tumoroids derived from *Apc^Min/+^ Stat2^−/−^* as compared with *Apc^Min/+^* WT mice as indicated by the results obtained in the MTT-formazan (Supplementary Figure S4C) and the propidium iodide/Hoechst assays (Supplementary Figure S5A,B).

Taken together, these experiments indicated that intestinal epithelial STAT2 can interfere with the actions of two different anti-cancer drugs which rely on different pathways to induce cell death.

## 4. Discussion

Although recent improvements in screening procedures caused a drop in the rates of advanced CRC in elderly patients, this trend has been obscured by the increasing incidences in patients aged 50 and below. Due to its complex pathogenesis, heterogeneous clinical manifestations, and limited therapeutic options for successfully treating advanced stages of the disease [45,46], CRC remains a medical challenge and overall one of the major socioeconomic burdens of our time [1,2]. We set out to understand the role played by STAT2, a key modulator of type I IFN signals, in the context of CRC. Using two independent mouse models relying on different pathogenetic mechanisms coupled with functional experiments in tumoroids, we could show that STAT2 promotes CRC and is implicated in the resistance of tumor cells to the anti-cancer agents 5-Fluorouracil and Cisplatin.

Accumulating evidence suggests that STAT2 may be a critical player in the tumor microenvironment [10] as it was shown to be able to either promote [29] or block [30,47] tumorigenesis. Very recent data indicated that STAT2 controlled the level of intestinal inflammation in different mouse models of experimental colitis. Importantly, epithelial STAT2 was shown to be important in that context since findings obtained with *Stat2^ΔICE^* mice (specifically lacking STAT2 in epithelial cells) recapitulated the main results observed in mice with germline knockout of STAT2 [22]. Many genes associated with the pathogenesis or the metastasis of CRC, e.g., *Gbp5* [48], *Hif3a* [49], *Hp* [50], *Il1f9/Il36g* [51], *Lrg1* [52], *Ups18* [53], as well as markers of poor-prognosis/therapy failure in CRC, e.g., *Cd14* [54], *Cdc20* [55], *Mmp8* [56], or *Mmp10* [57] were present among the top most downregulated transcripts in *Stat2^−/−^* mice receiving DSS, as compared to WT mice from the DSS model [22]. These RNA-Seq data strongly suggested that STAT2 might be implicated in the control of tumorigenesis in the colon. To address this experimentally, we subjected WT and *Stat2^−/−^* mice to AOM–DSS experiments. Tumors appeared earlier and were bigger in WT mice which also presented more intestinal inflammation as indicated by the mini-endoscopy and histologic evaluation of colon sections. Notably, a few bigger tumors were also found in STAT2-deficient mice upon post-mortem examination of colons. The mechanistic details by which STAT2 controls the initiation (reflected by the tumor number) and the progression (reflected by tumor size) phases of tumorigenesis [32] remain to be addressed by future studies.

Furthermore, increased levels of pSTAT3 were present in more susceptible WT as compared with *Stat2^−/−^* mice. Our findings are in line with previously published results obtained using alternative protocols for the induction and evaluation of AOM–DSS [29]. To explore the implication of STAT2 signaling beyond the inflammation-driven CRC, we employed the non-inflammatory, genetically-based model of *Apc^Min/+^* mice. Recapitulating the main findings from the AOM–DSS model, *Stat2^−/−^* mice on the *APC^min/+^* background developed significantly fewer tumors which appeared later and were overall smaller than those developed by *Apc^Min/+^* WT mice. Tumors from *Apc^Min/+^ Stat2^−/−^* mice presented lower levels of STAT3, phosphorylated STAT3, and cyclin D1, a downstream target of STAT3 signaling. Importantly, increased expression of cyclin D1 was shown to be an early event in the development of CRC [58]. Taken together, our in vivo data indicated that STAT2 promotes CRC by inflammation-dependent and inflammation-independent mechanisms, and suggested that STAT3/cyclin D1 might be implicated in this process. Noteworthy, consulting publicly available datasets, we found a significant correlation between the expression levels of *STAT2* and *STAT3* as well as *STAT2* and cyclin D1 in samples from patients with CRC.

We next wanted to understand whether the effects observed in the experimental mouse models rely on the function of STAT2 in epithelial cells. Therefore, we isolated individual tumors from *Apc^Min/+^* WT and *Apc^Min/+^ Stat2^−/−^* mice and cultured them as three-dimensional tumoroids. Interestingly, although tumoroids from both strains could be readily produced, there was a clear difference in the phenotype of developing tumoroids in the sense that *Apc^Min/+^* WT tumoroids grew faster and bigger as compared with *Apc^Min/+^ Stat2^−/−^* tumoroids. Moreover, morphologic changes that are typically characteristic of differentiating and dying organoids (e.g., thickening of the wall, darkening and accumulation of dead cells in the lumen, shedding of dead cells, budding) were present at much higher rates in tumoroids generated from tumors of *Apc^Min/+^ Stat2^−/−^* mice as compared to those from *Apc^Min/+^* WT mice. This observation is in stark contrast to the fact that in the absence of the *Apc^Min/+^* mutation, organoids generated from the small intestine and the colon of *Stat2^−/−^* mice are more robust compared with organoids generated from WT control mice [22]. Whether STAT3 plays a role in this context is not known, but in the present study, the levels of pSTAT3 and STAT3 were found to be increased, although not significantly, in the non-tumor tissue from *Apc^Min/+^ Stat2^−/−^* as compared to the non-tumor tissue from *Apc^Min/+^* WT mice (refer to Supplementary Figure S2E). It remains to be addressed by further studies, using tissue-specific conditional deleters, how various cell types employ STAT2 and STAT3 signals to control discrete functions in normal (i.e., organoid) vs. cancer (i.e., tumoroid) epithelium. The morphologic differences observed in our tumoroids were further confirmed by results of quantitative polymerase chain reaction which indicated significantly higher levels of stem cell/cancer stem cell markers (e.g., *Lgr5*, *Axin2*, *Cd33*, *Prom1/Cd133*) and lower levels of the differentiation marker *Muc2* in *Apc^Min/+^* WT tumoroids as compared with *Apc^Min/+^ Stat2^−/−^* tumoroids. The relevance of these findings is further underlined by the fact that high levels of CD133 [59] and low levels of MUC2 [60] represent poor prognostic markers for CRC. The fact that phosphorylated STAT3 and cyclin D1 were also downregulated in STAT2-deficient tumoroids indicated that the differences observed in vivo might rely on the activity of STAT2 in epithelial cells.

The relapse-free survival period achievable through anti-cancer medication is substantially reduced for undifferentiated cancers. Since tumoroids derived from *Apc^Min/+^ Stat2^−/−^* were better differentiated than tumoroids from *Apc^Min/+^* WT, we next wondered whether anti-cancer drugs would be more efficient in killing the former. Treatment with 5-Fluorouracil killed tumoroids in a dose- and time-dependent manner. Importantly, differentiated *Apc^Min/+^ Stat2^−/−^* were more susceptible as compared with *Apc^Min/+^* WT tumoroids. It is noteworthy to mention the fact that LGR5 overexpression, as observed in *Apc^Min/+^* WT tumoroids (refer to Figure 3E), has been previously linked to resistance to 5-Fluorouracil-based chemotherapy in CRC [61]. In complementary experiments, we used Cisplatin as the anti-cancer drug. Treating tumoroids with 100 µM Cisplatin resulted in the killing of the vast majority of *Apc^Min/+^ Stat2^−/−^* after two days and virtually all of them after four days. Comparatively, *Apc^Min/+^* WT tumoroids were significantly more resistant. The anti-tumor activity of Cisplatin could be boosted by increasing the nuclear accumulation of HMGB1 protein, an inhibitor of Cisplatin DNA-adduct repair [62]. On the other hand, the extracellular release of HMGB1 accompanied apoptotic and necroptotic cell death in Cisplatin-susceptible lung adenocarcinoma cell lines [63]. To get a first glimpse of how STAT2 signaling might interfere with this, we measured levels of HMGB1, an indirect marker of DNA damage, in supernatants of treated tumoroids. The results indicated that the absence of STAT2 was associated with an increased excretion of HMGB1. The difference between the DNA damage response in the two mouse strains was further underlined by the fact that Cisplatin preferentially induced DNA fragmentation in the more sensitive *Apc^Min/+^ Stat2^−/−^* compared with more resistant *Apc^Min/+^* WT tumoroids. There is great hope that genome-wide three-dimensional multi-omics approaches that enable functional studies on the role of chromatin structure modification in the development of CRC and the resistance to anti-cancer drug therapy will play a decisive role in the future management of the disease [14]. Furthermore, magnetic micro-/nano-robot multimers have recently been shown to possess great potential for enhanced targeted anti-cancer drug delivery systems [15]. Taken together, our present results indicated that two anti-cancer drugs which induce cell death by different mechanisms [43,44], preferably kill highly differentiated STAT2-deficient tumoroids. Novel biomarkers for both early diagnosis and patient stratification based on precisely defined molecular patterns for the appropriate therapy are needed to improve survival rates in CRC patients in the context of personalized oncology. It is therefore conceivable that lowering STAT2 function, e.g., by dietary phytoestrogens [64], in poorly differentiated tumors, might render them more susceptible to killing by anti-cancer drugs. A recent meta-study found that increased tumor infiltration with Th1 and CD8^+^ T cells was associated with the expression status of STAT2 and its target CXCL10, suggesting that the activation of this pathway could predict the outcome in oral cancer patients [47].

Various scenarios may explain how a deficiency in STAT2 protects from CRC. First, IRF9 and unphosphorylated STAT2 have been shown to cooperate with NF-κB to drive the expression of IL-6 [65], which can promote STAT3-dependent CRC growth [66]. This hypothesis might be in part responsible for our observations, since STAT3 and its downstream target, cyclin D1, were downregulated in mice lacking STAT2. A further piece of evidence strengthening this hypothesis is provided by the fact that compared with *Apc^Min/+^* WT mice, *Apc^Min/+^ Stat2^−/−^* mice showed decreased levels of the specific NF-κB target *Il6*, whereas general inflammation markers were not altered (refer to Supplementary Figure S2C,D). Second, STAT2 might co-orchestrate together with IRF9, and to a lesser extent STAT1, the expression of a subset of interferon-related DNA damage signature genes that have been previously associated with resistance to chemotherapy and radiotherapy [67,68,69,70]. Third, STAT2-dependent inhibition of STING promoted chemotherapy resistance and tumor progression in a study reporting a significant association between high STAT2 tumor levels and shorter overall survival in patients with lung adenocarcinomas [71]. The fact that *Apc^Min/+^* WT tumoroids were resistant to anti-cancer drugs provides circumstantial evidence for the second and third scenarios, as well. The intricate mechanisms through which STAT2-mediated signals control resistance to anti-cancer drugs have not been further addressed in our study, and thus remain a subject for future endeavors. Given the fact that STAT2-dependent signaling mediates a highly conserved biological response in humans and mice, it is conceivable that our results might represent a solid basis for translational approaches focused on targeting this pathway in patients with CRC.

## 5. Conclusions

Our findings demonstrate for the first time that STAT2 promotes CRC through inflammation-driven and inflammation-independent mechanisms. Importantly, the lack of STAT2 increased the susceptibility of intestinal tumoroids to cell death induced by different anti-cancer drugs that kill by engaging non-overlapping molecular mechanisms. The use of recently produced *Stat2*-conditional knockout mice will be instrumental in addressing the precise nature of STAT2-dependent signals in tumor cells and the tumor microenvironment. Taken together, the results of the present study suggest that reducing STAT2 signaling in colorectal tumors may potentially boost therapy responses.

## Data Availability

Data related to the project are presented in this article. RNA-Seq data are deposited on ArrayExpress E-MTAB-12655.

## Supplementary Materials

The following supporting information can be downloaded at: https://www.mdpi.com/article/10.3390/cancers15225423/s1, Figure S1: Schematic illustration of the possible mechanisms by which STAT2 could control CRC; Figure S2 Inflammation-independent role of STAT2 in the *Apc^Min/+^* model in vivo and ex vivo; Figure S3: Effects of Cisplatin on the DNA damage in *Apc^Min/+^* tumoroids; Figure S4: 5-Fluorouracil induces cell death in *Apc^Min/+^* tumoroids in a dose- and time-dependent manner; Figure S5: *Apc^Min/+^* tumoroids lacking STAT2 have a higher susceptibility to the induction of cell death by 5-Fluorouracil as compared with *Apc^Min/+^* WT tumoroids; Figure S6: Source of western blots.
