# Supplementary material for: STAT2 Controls Colorectal Tumorigenesis and Resistance to Anti-Cancer Drugs
Source: Cancers (Basel). 2023 Nov 15;15(22):5423. doi: 10.3390/cancers15225423 (PMC10670206; doi:10.3390/cancers15225423)
Supplement: Supplementary file 1 [file cancers-15-05423-s001.zip › cancers-2683582-supplementary.pdf]

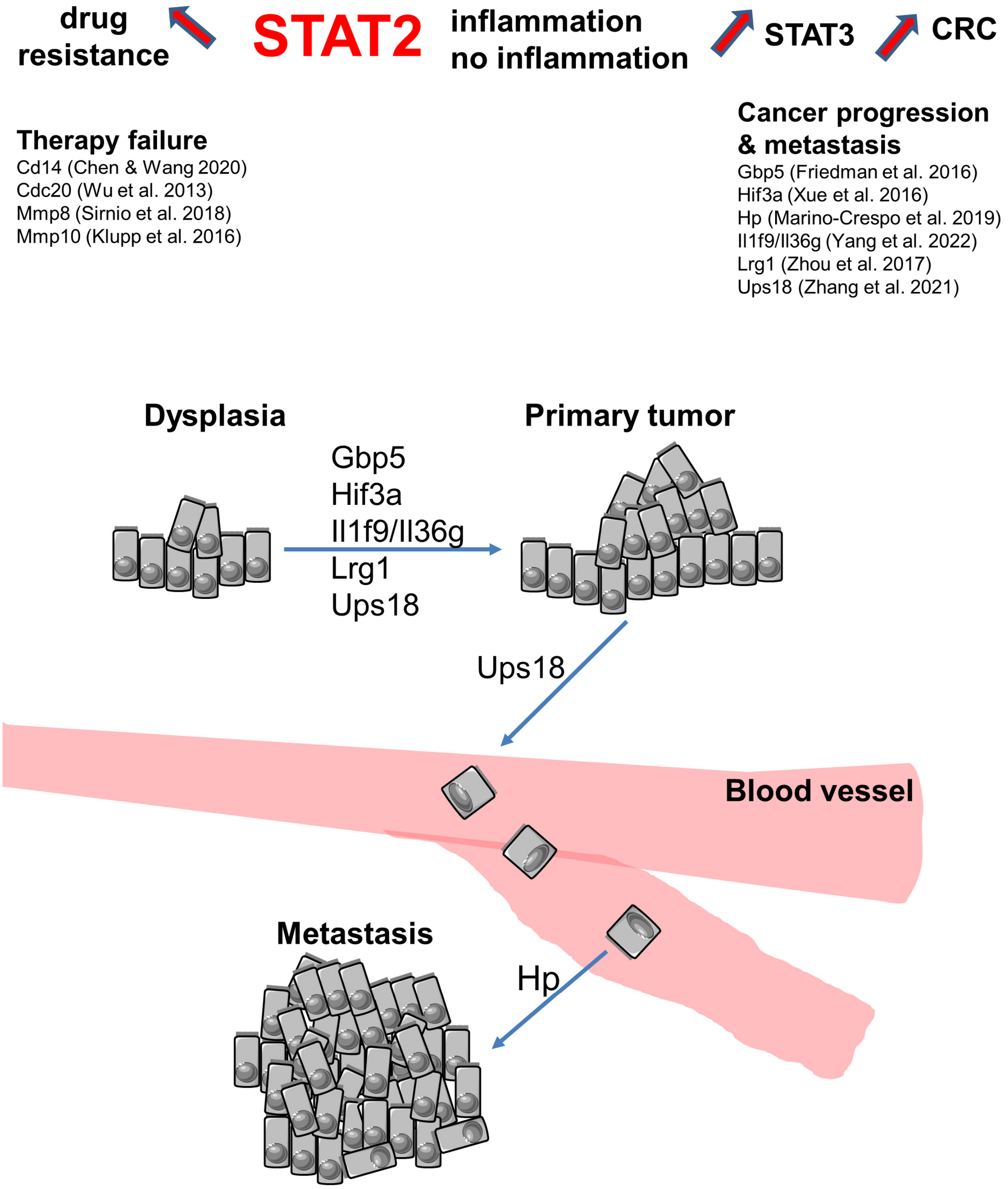

Supplementary Figure S1. Schematic illustration of the possible mechanisms by which STAT2 could control CRC.

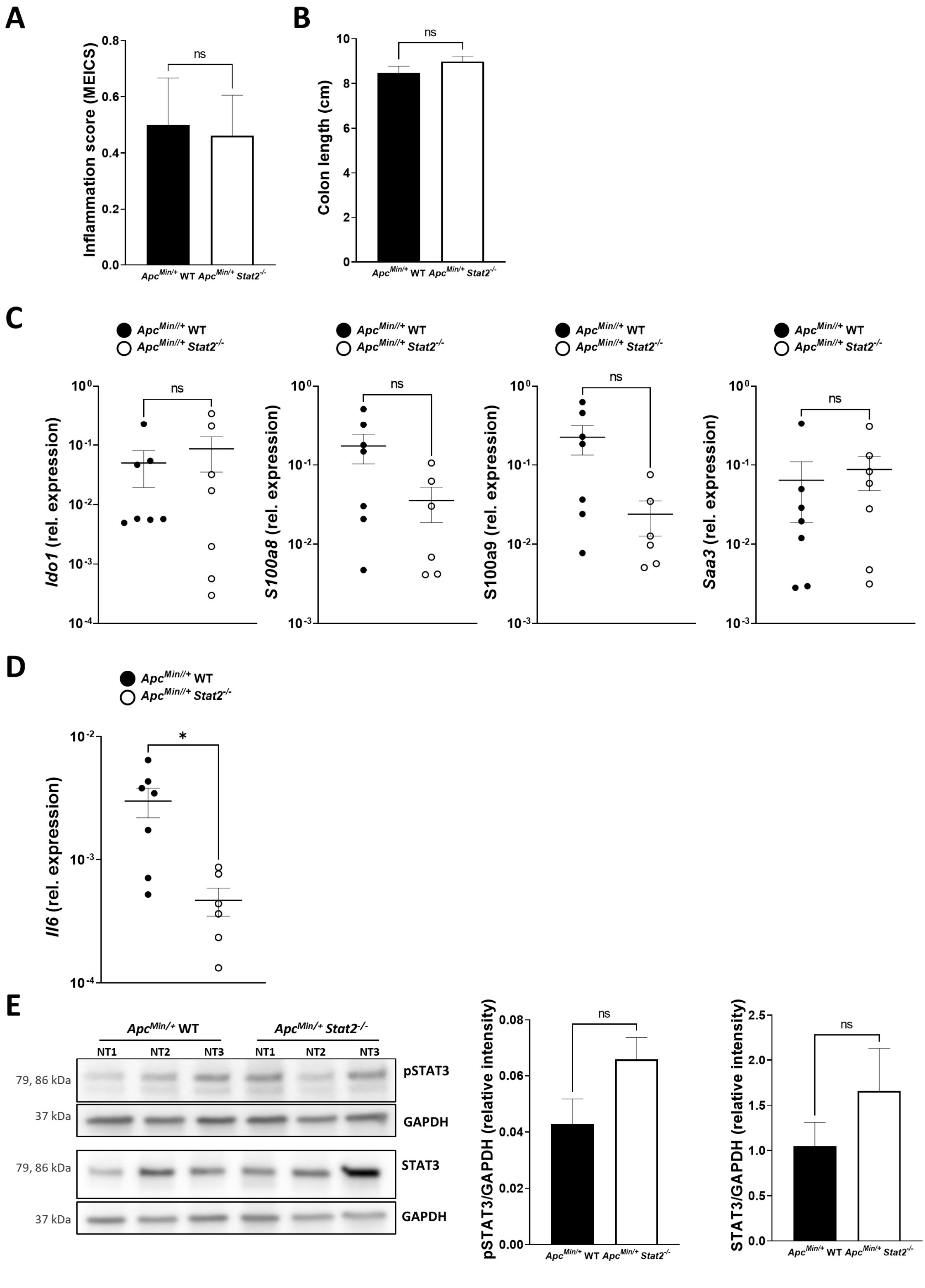

Supplementary Figure S2. Inflammation-independent role of STAT2 in the ***Apc$^{Min/+}$*** **model** ***in vivo*** **and** ***ex vivo.*** Inflammation in WT and *Stat2*-deficient mice on the *Apc$^{Min/+}$* background was investigated by endoscopy (MEICS score in **A**) and the length of colons (**B**). (**C**) Expression levels of typical inflammation markers in the *Apc$^{Min/+}$* model of CRC were determined by quantitative polymerase chain reaction. (**D**) The expression levels of the specific NF-kB target gene *Il6* in *Apc$^{Min/+}$* WT and *Apc$^{Min/+}$ Stat2$^{-/-}$* mice were investigated by quantitative polymerase chain reaction. (**E**) Western blot analysis of pSTAT3 and STAT3 levels in non-tumor tissue samples from *Apc$^{Min/+}$* WT and *Apc$^{Min/+}$ Stat2$^{-/-}$* mice. GAPDH band intensity was used as a loading control. Quantification of the normalized values. Abbreviation: ns, not significant, NT, non-tumor. Statistics: Welch's *t*-test in A, B, and E and Mann-Whitney in C and in D; mean with SEM is displayed.

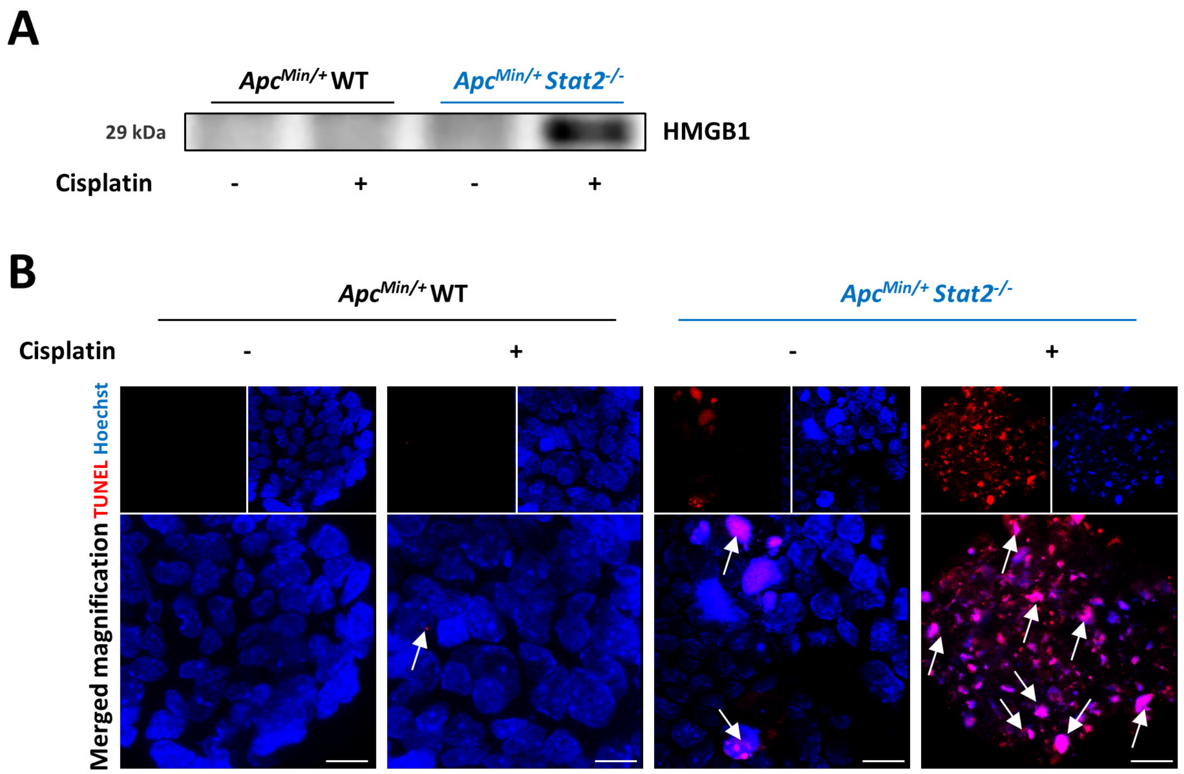

Supplementary Figure S3. Effects of Cisplatin on the DNA damage in *Apc*$^{Min/+}$ tumoroids. (**A**) The levels of extracellularly released HMGB1 were measured by western blot in supernatants of tumoroids cultured in the presence of Cisplatin. Wells receiving only vehicle served as controls. (**B**) Confocal images of DNA fragmentation evaluated by the TUNEL assay in tumoroids from the Cisplatin stimulation experiments; positive cells are indicated by arrows. Hoechst was used to counterstain nuclei. Scale bars, 10μm.

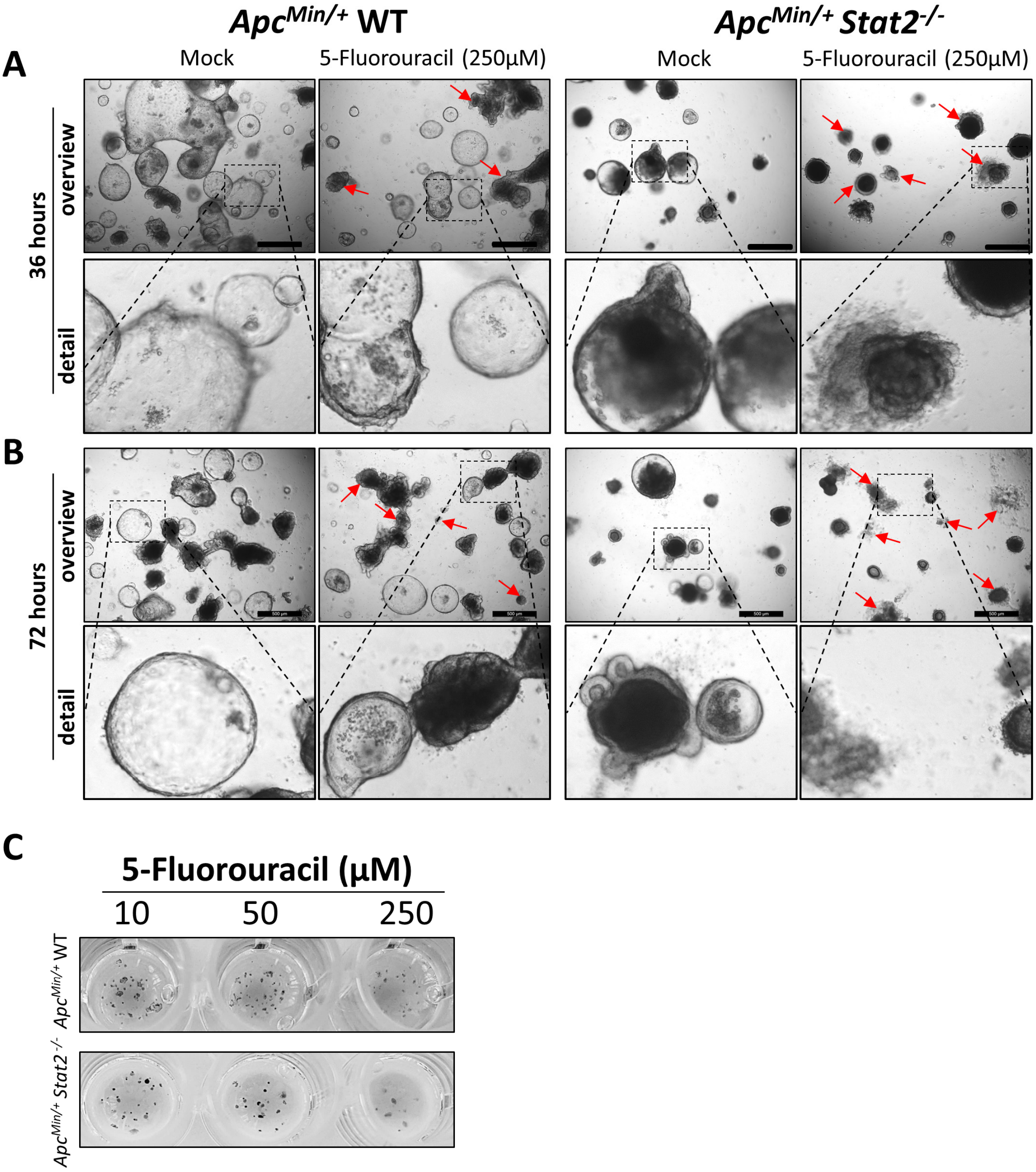

Supplementary Figure S4. 5-Fluorouracil induces cell death in *Apc*$^{Min/+}$ tumoroids in a dose- and time-dependent manner. (**A**) Representative light microscopy images from an experiment in which the effect of the highest dose of 5-Fluorouracil used in our study, namely 250µM, was comparatively followed in tumoroids derived from *Apc*$^{Min/+}$ WT and *Apc*$^{Min/+}$ *Stat2*$^{-/-}$ mice after (**A**) 36 hours and (**B**) 72 hours. Red arrows in A and B indicate highly differentiated/dead tumoroids. Scale bars, 500µm. (**C**) Macroscopic images indicating the presence of formazan precipitates as black spots of different sizes in the MTT-formazan assay. The concentrations of the anti-cancer drug used in the assay are indicated above the images of the wells.

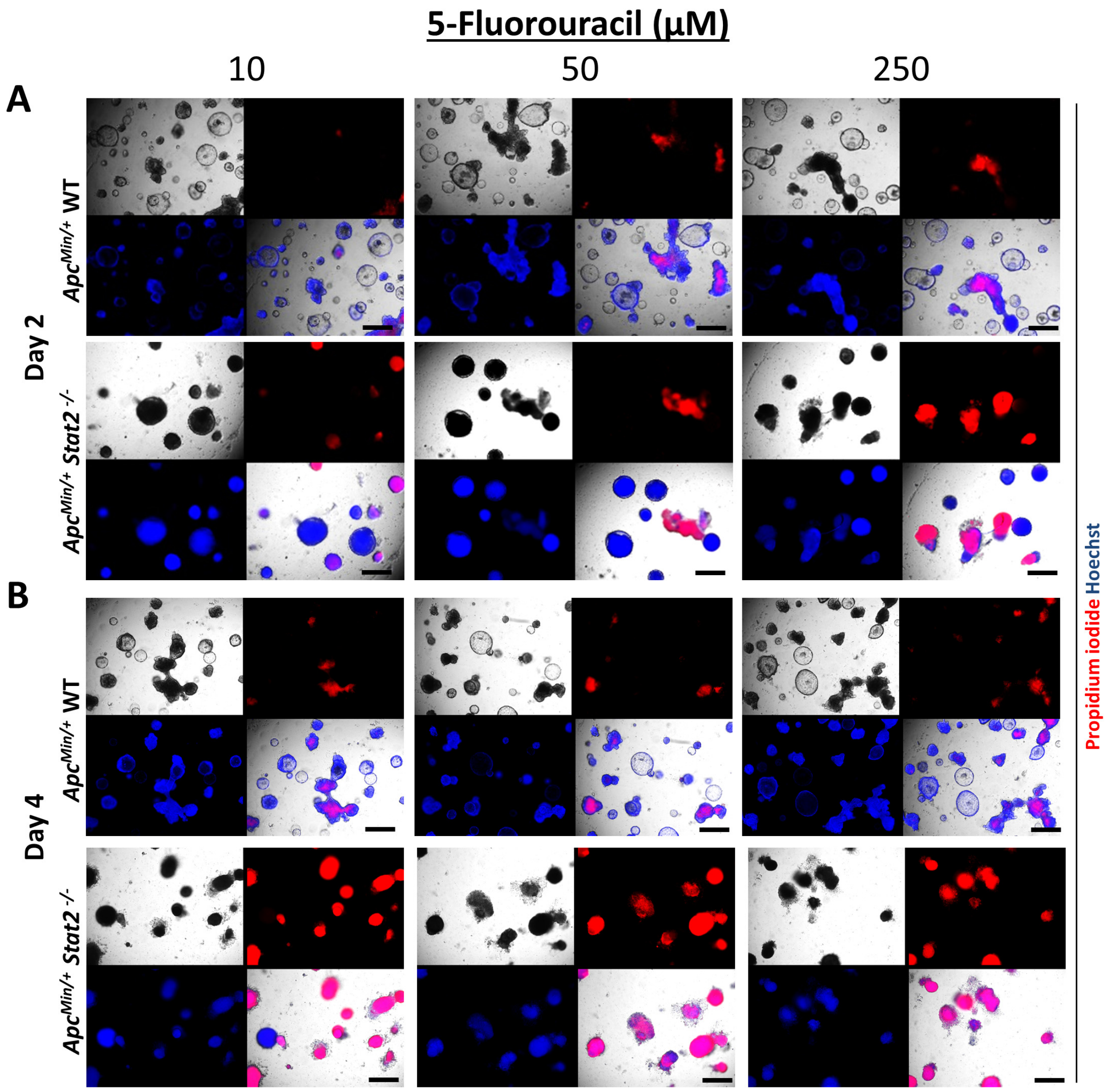

Supplementary Figure S5. ***$Apc^{Min/+}$*** **tumoroids lacking STAT2 have a higher susceptibility to the induction of cell death by 5-Fluorouracil as compared with** ***$Apc^{Min/+}$*** **WT tumoroids.** (**A, B**) Induction of cell death by three different doses of 5-Fluorouracil was evaluated by fluorescence microscopy after co-staining of *$Apc^{Min/+}$ $Stat2^{-/-}$* and *$Apc^{Min/+}$* WT tumoroids with propidium iodide (red staining - dead cells) and Hoechst (blue staining – all cells) at an early (two days) and a late (four days) time point. Scale bars, 500µm.

Source of western blots used in:

Figure 2E

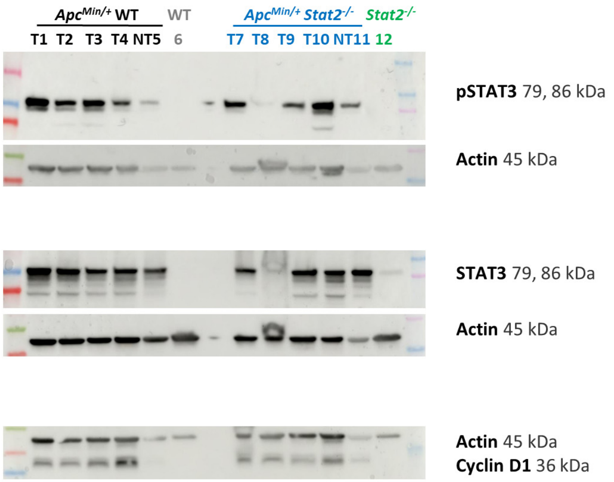

Suppl. Figure 2E

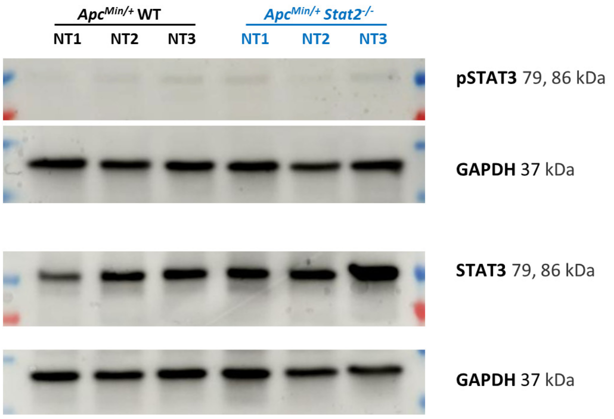

Suppl. Figure 3A

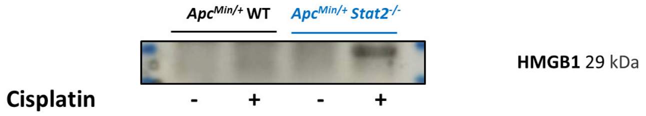

Supplementary Figure S6. Source of western blots.
